# Supplementary material for: Role of Polyunsaturated Fatty Acids (PUFAs) and Eicosanoids on Dry Eye Symptoms and Signs
Source: Biomolecules. 2024 Mar 20;14(3):376. doi: 10.3390/biom14030376 (PMC10968634; doi:10.3390/biom14030376)
Supplement: Supplementary file 1 [file biomolecules-14-00376-s001.zip › biomolecules-2889426-supplementary.pdf]

biomolecules

MDPI

Supplemental Table S1. Median and ranges for tear lipids collected.

Pro-Inflammatory					Anti-Inflammatory				Pro:anti-Inflammatory Ratios		
Median (min-max)											
**AA***	**TXB_2**	**5-HETE**	**12-HETE**	**15-HETE**	**DHA**	**EPA**	**11,12 DHET**	**14,15 DHET**	**AA: DHA**	**AA: EPA**	**ω6:ω3**
629.6 (65.0–4921.7)	12.1 (0.0–149.8)	0.8 (0.1-4.3)	2.9 (0.2–13.7)	1.3 (0.1-12.5)	82.4 (11.9–480.2)	9.1 (0–95.7)	0.0 (0.0–0.2)	0.1 (0.0–0.4)	7.0 (4.1–15.2)	64.1 (0–1365.1)	6.5 (3.0–12.7)
Percentage (%) recovered											
100%	58%	100%	100%	100%	100%	95%	68%	85%	100%	95%	100%

AA: Arachidonic acid; **TXB_2**: Thromboxane B2; **5-HETE**: 5-hydroxyeicosatetraenoic acid; **12-HETE**: 12-hydroxyeicosatetraenoic acid; **15-HETE**: 15-Hydroxyeicosatetraenoic acid; **DHA**: Docosahexaenoic acid; **EPA**: Eicosapentaenoic acid; 11,12 **DHET**: 11,12-dihydroxyeicosatrienoic acid; 14,15 DHET: 14,15-dihydroxyicosatrienoic acid; ω6: Omega 6; ω3: Omega 3. * Unit of each lipid is normalized with the amount of protein in the sample as a pico mole of lipid per milligram of protein [pmol/mg protein].
